# Human Mesenchymal Stem Cells Display Reduced Expression of CD105 after Culture in Serum-Free Medium

**DOI:** 10.1155/2013/698076

**Published:** 2013-09-30

**Authors:** Peter Mark, Mandy Kleinsorge, Ralf Gaebel, Cornelia A. Lux, Anita Toelk, Erik Pittermann, Robert David, Gustav Steinhoff, Nan Ma

**Affiliations:** Reference and Translation Center for Cardiac Stem Cell Therapy (RTC), University of Rostock, Schillingallee 68, 18057 Rostock, Germany

## Abstract

Human Mesenchymal Stem Cells (hMSCs) present a promising tool for regenerative medicine. However, *ex vivo* expansion is necessary to obtain sufficient cells for clinical therapy. Conventional growth media usually contain the critical component fetal bovine serum. For clinical use, chemically defined media will be required. In this study, the capability of two commercial, chemically defined, serum-free hMSC growth media (MSCGM-CD and PowerStem) for hMSC proliferation was examined and compared to serum-containing medium (MSCGM). Immunophenotyping of hMSCs was performed using flow cytometry, and they were tested for their ability to differentiate into a variety of cell types. Although the morphology of hMSCs cultured in the different media differed, immunophenotyping displayed similar marker patterns (high expression of CD29, CD44, CD73, and CD90 cell surface markers and absence of CD45). Interestingly, the expression of CD105 was significantly lower for hMSCs cultured in MSCGM-CD compared to MSCGM. Both groups maintained mesenchymal multilineage differentiation potential. In conclusion, the serum-free growth medium is suitable for hMSC culture and comparable to its serum-containing counterpart. As the expression of CD105 has been shown to positively influence hMSC cardiac regenerative potential, the impact of CD105 expression onto clinical use after expansion in MSCGM-CD will have to be tested.

## 1. Introduction

Human MSCs play an essential role in today's medical research, as they promise new approaches in treatment of human diseases. Their plasticity and immense *ex vivo* proliferation potential make MSC an important tool for cell transplantation as well as generation of living, functional tissue suitable for organ repair and replacement. Mesenchymal stem cells have already been successfully applied in treatment of *osteogenesis imperfecta* [1] to limit graft-versus-host disease via immunosuppression [2]. Furthermore, it has been reported that MSCs contribute to the regeneration process after myocardial infarction in mice [3] and are able to improve the outcome of allogeneic transplantation in general by means of immunomodulatory effects [4]. MSCs were first described as plastic adherent, clonogenic, colony-forming fibroblast-like cells [5]. So far, no generally accepted definition of MSC exists, although they are identified by specific properties [6]. The most important is the hMSC ability to self-renew and to give rise to mature cells of adipogenic, osteogenic, and chondrogenic lineage, producing tissues such as bone, cartilage, tendon, adipose tissue, and hematopoietic supporting stroma [7–9]. 

The expression of cell surface markers such as CD29, CD44, CD90 [9], CD73, and CD105 [10] allows a fast characterization of hMSCs. Bone marrow-derived hMSCs represent nonhematopoietic stem cells and therefore lack typical hematopoietic surface antigens like CD14, CD34, and CD45 [11]. CD105 (Endoglin) is a membrane glycoprotein and part of the transforming growth factor-*β* receptor complex. It plays an important role in angiogenesis [12]. Our own previous studies demonstrated that after myocardial infarction in mice transplantation of hMSCs with high CD105 expression is crucial for significantly improved myocardial performance.

Human bone marrow aspirates of voluntary donors typically contain hMSCs in numbers too small for clinical application. Therefore, *in vitro* expansion is necessary. To maintain multipotency of proliferating hMSCs conditions similar to the in vivo situation are necessary. This is in general achieved by addition of bovine serum, which comprises growth factors, hormones, amino acids, proteins, and other components required for cell proliferation *ex vivo*. Studies revealed human serum to be less effective than fetal bovine serum (FBS or FCS), which is therefore usually applied [13]. Unfortunately, bovine-derived serum components may give rise to immune reactions by transplant recipients [14]. For clinical use, *ex vivo* expanded hMSCs still represent a dangerous health risk, because they retain FCS-derived components. Others reported that hMSC expansion in autologous serum is as effective as supplementing the cultures with FBS [15]. However, autologous peripheral blood provides volumes too small to be of practical relevance [16]. The use of pooled allogeneic serum has been attempted as well but was demonstrated to result in cell arrest and even cell death and is therefore also not suitable for therapeutic use [17].

In order to provide serum-free culture conditions, Müller and coworkers successfully replaced FCS by human platelet lysate and fresh frozen plasma [18]. Still, some disadvantages remain, like undefined and inconstant composition of used plasma/serum, complicating the supply of reproducible formulations of medium. An additional risk lies in possible contamination of serum with bacteria, viruses, or mycoplasma [19]. Finally, limited availability and cost of serum must not be ignored. Suitable chemically defined growth media that abolish the necessity of serum addition would solve the issues of limited clinical use of hMSCs today.

One commercial serum-free medium (UC supplemented with ULTROSER) has been demonstrated to achieve better cell expansion than animal serum-containing medium [20]. However, it is important to extensively test every serum-free medium for its capacity to expand hMSCs and clinical relevance. Recently, several new commercially available, hMSC specific, serum-free growth media reached market maturity. We tested two commercially available growth media (MSCGM-CD and PowerStem) for their ability to promote hMSC proliferation in comparison to conventional serum-containing medium. 

The aim of our study was to analyze (i) whether serum-free cultured hMSCs express typical surface markers and (ii) if they maintain hMSC plasticity, evaluated by their capacity to differentiate into adipogenic, osteogenic, and chondrogenic progenitors. This was examined in comparison to hMSCs cultured in conventional, serum-containing growth medium. One serum-free culturing condition (MSCGM-CD) provided reliable expansion support and was therefore further investigated regarding surface marker expression and differentiation potential compared to cells grown in MSCGM.

## 2. Materials and Methods

### 2.1. Patient Samples

 In all experiments, sternal bone marrow aspirates of voluntary donors was used after their consent was obtained according to ethical approval of the local ethical committee of the University of Rostock and has been performed in accordance with the ethical standards of the Declaration of Helsinki. Patients were 64 (±12) years old and underwent open heart surgery. Seven of them were males, and three were females (See Figure S1 in Supplementary Material available online at http://dx.doi.org/10.1155/2013/698076). Thereby, none of the patients either abused alcohol or consumed tobacco. However, four out of the ten patients had consumed tobacco in the past (Figures S1 and S2). The average BMI of the patients was 27.79 ± 5.15 (kg/m²) which is why they are in average considered as preobese.

### 2.2. Cell Culture

Human bone marrow samples of patients were divided into portions of 7 mL which were subsequently seeded into either serum-free MSGCM-CD (Lonza Group AG, Cologne, Germany), PowerStem (PAN Biotech GmbH, Aidenbach, Germany), or serum-containing medium MSCGM (Lonza Group AG). Heparinized (250–500 I. U.) human bone marrow aspirate was transferred into cell culture flasks (75 cm², Greiner Bio-One GmbH, Frickenhausen, Germany). The flasks were stored at 37°C and 5% CO_2_. Regular medium changes were performed every 2-3 days.

### 2.3. Cell Passage

After bone marrow seeding, 10–16 days of primary culture (=passage 0) were required until hMSC colonies reached confluency. At this time point, splitting was performed. Thereby, 5000–7000 cells per cm² were seeded into new culture flasks as passage 1. After reaching confluency, the procedure was repeated up to passage 3. From passages 1 to 3, splitting periods were three to nine days. Thereby, the mean doubling time in the different media ranged from 2.60 to 4.00 days (see Section 3).

In detail, splitting procedures were as follows: layers were washed with prewarmed PBS (PAN Biotech GmbH) and passaged by the addition of trypsin (2.5 mg/mL, 1 mL per 25 cm² of cell culture surface, PAA Laboratories GmbH, Cölbe, Germany) to detach the cells. Previously removed medium was added as trypsin inhibition. As serum-free MSCGM-CD and PowerStem alone did not seem sufficient for trypsin inhibition, 1.5 mL HSA (Human albumin 20%, low salt; CSL Behring GmbH, Hattersheim Main, Germany) was added to the cells cultured in this medium. The whole suspension was centrifuged (300 ×g, 20°C, 10 min). The supernatant was discarded, and the remaining cell pellet was resuspended in 0.5–2 mL of appropriate medium for cell counting. Human MSCs were analysed in differentiation assays and fluorescence-activated cell sorting (FACS) after third passage.

### 2.4. Differentiation Assays

To examine the multilineage potential of cultured hMSCs, they were differentiated into adipogenic, osteogenic, and chondrogenic lineages using the “Human Mesenchymal Stem Cell Functional Identification Kit” (R&D Systems, Wiesbaden-Nordenstadt, Germany) according to the manufacturer's protocol. Human MSCs were seeded on cover slips (Menzel GmbH, Braunschweig, Germany) in 24-well plates (Greiner Bio-One) for adipogenic (37000 cells per well) and osteogenic (7400 cells per well) differentiation, respectively, while cell pellets (250000 cells per pellet) were used for chondrogenic differentiation. When cells for adipogenesis were 100% confluent and cells for osteogenesis were 50%–70% confluent, they were induced with appropriate differentiation medium, which was changed every 3-4 days (R&D Systems). Cells for chondrogenesis were seeded directly in chondrogenic differentiation medium with medium changes every 2-3 days (R&D Systems). After 21 days, all cells were fixed in 2% paraformaldehyde solution (Sigma-Aldrich Chemie GmbH, Munich, Germany), and chondrogenic pellets were embedded in Tissue-Tek mounting medium (Sakura Finetek Europe B.V., Alphen aan den Rijn, Netherlands) and cryosectioned into 5 *μ*m thick slices.

### 2.5. Immunocytochemical Staining

The differentiated hMSCs were stained immunocytochemically in order to analyze their commitment to adipogenic, osteogenic, or chondrogenic lineages. The staining was performed according to the manufacturer's protocol of the “Human Mesenchymal Stem Cell Functional Identification Kit” (R&D Systems). Cells were permeabilized with 0.5 mL of 1x PBS containing 0.3% Triton X-100 (Carl Roth GmbH + Co. KG, Karlsruhe, Germany), blocked for 45 min with 1% HSA and 10% horse serum (PAA Laboratories GmbH) in 1x PBS at room temperature. Cells were incubated with appropriate primary antibody (1 : 350 in 1% HSA, 10% horse serum) overnight at 4°C, or without primary antibody serving as negative control. Primary antibodies (R&D Systems) were directed against FABP-4 for adipogenic, osteocalcin for osteogenic, and against aggrecan for chondrogenic differentiation. Cells were washed with 1x mL PBS (1% HSA) and incubated for staining with 250 *μ*L of adequate 1 : 300 diluted secondary antibody (AlexaFluor488 anti mouse, Invitrogen, Life Technologies Corporation, Carlsbad, USA) for 60 min at room temperature in the dark. After subsequent washing, 4′,6-diamidino-2-phenylindole (DAPI, Invitrogen) was used to stain cell nuclei (250 *μ*L of a 0.3 *μ*M DAPI solution for 10 min). Afterwards, cells were washed with 1x PBS containing 1% HAS and distilled water. Finally, cells or cryosections were mounted on glass slides using prewarmed (60°C) Dako glycergel mounting medium (Dako GmbH, Hamburg, Germany). 

### 2.6. Fluorescence Microscopy Analysis

Fluorescence microscopy images of hMSCs were taken after differentiation assays and staining. To determine the rate of cells responding to the given stimuli, cells were counted in 10 high power fields that contained at least 100 cells, and the number of cells stained positive for the differentiation marker was compared with the amount of nuclei (representing total amount of cells). Confocal laser scanning microscopy (ELYRA, Carl Zeiss AG, Jena, Germany) was used to obtain high resolution images of immunocytochemically stained differentiated hMSCs at magnifications 100x and 400x.

### 2.7. Fluorescence-Activated Cell Sorting (FACS)

During the whole staining procedure cells, antibodies and all reagents were kept on ice. Biotinylated antibodies were prepared by mixing 4 *μ*L of anti-CD90-biotin and 40 *μ*L of biotin-isotype control (Table 1) with 1 *μ*L streptavidin-V450 (BD Biosciences), respectively. Mixtures were incubated at 4°C for 30 min. The required antibodies (Table 1) and 20 *μ*L FcR-block (BD Biosciences) were added to 100,000 cells in 1x PBS with 0.5% BSA and 2 mM EDTA. To achieve a final volume of 100 *μ*L 1x PBS with 0.5% BSA and 2 mM EDTA was added. An unstained control, single antibody stainings, an isotype control staining for each antibody, and an eight-color antibody staining were prepared. After an incubation of 30 min, the stained cells were washed with 1 mL PBS and spanned down (300 ×g, 4°C, 5 min). The cell pellet was resuspended in 150 *μ*L 1x PBS and transferred to FACS tube (BD Biosciences) for measurement. Data analyses were performed using FACSDiva software (BD Biosciences).

### 2.8. Statistical Analysis

For statistical analysis, the unpaired two-tailed Student's *t*-test using the software GraphPad Prism 5.0 was performed. Differences were significant if the *P* value of the *t*-test was ≤0.05.

## 3. Results and Discussion

### 3.1. Human MSCs Are Readily Expanded in Serum-Free and Serum-Containing Growth Medium

**Figure 1:**
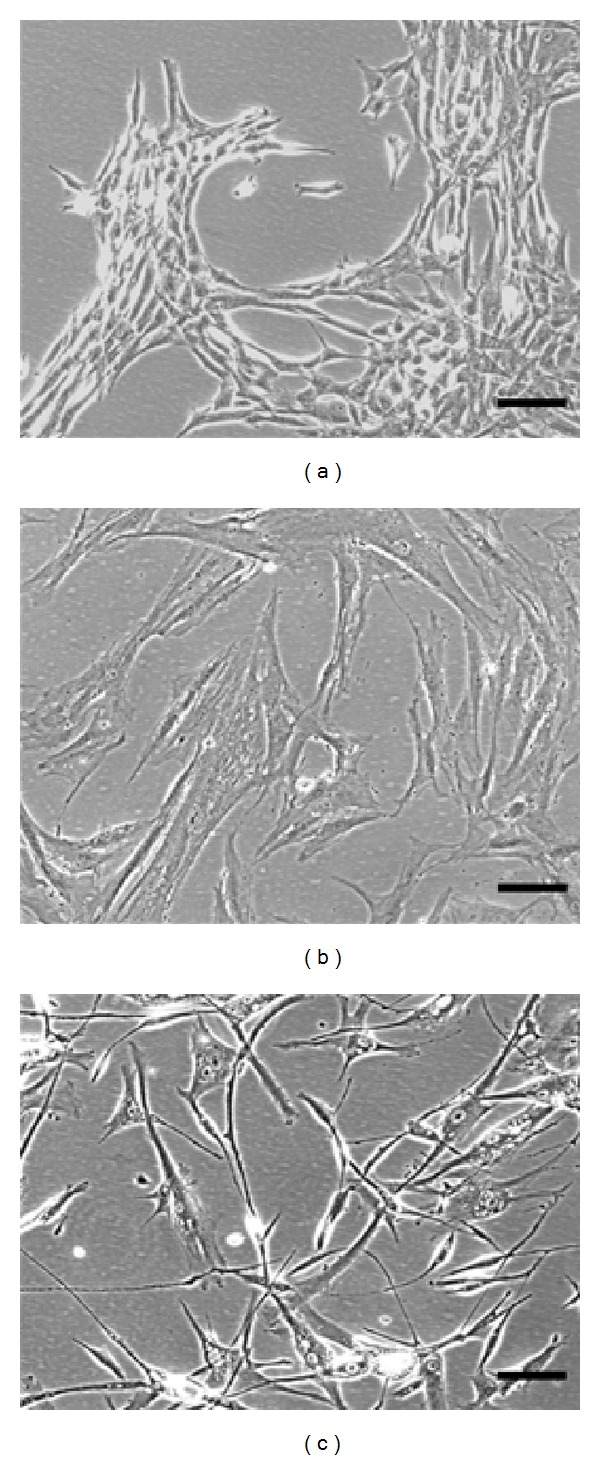
Morphology of hMSC colonies. Morphology of subconfluent hMSC colonies at day 8 cultivated in either MSCGM-CD (a), MSCGM (b), or PowerStem (c). 7 mL of bone marrow per donor were seeded in respective media. Representative pictures have been taken at 100x magnification. Scale bar (black) length = 100 *μ*m.

**Figure 2:**
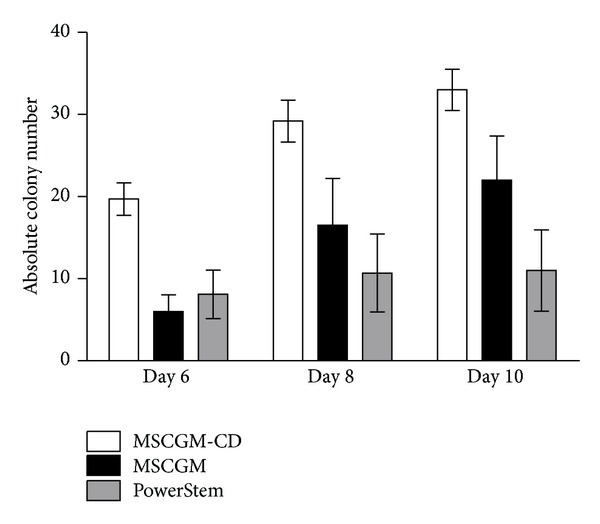
Quantity of hMSC colonies. Quantity of hMSC colonies at days 6, 8, and 10 grown in MSCGM (black), MSCGM-CD (white), and PowerStem (grey) growth medium, respectively. Data presented as mean ± SEM (*n* = 10). Unpaired two-tailed *t*-test was performed for statistical analysis. Differences were not significant between groups.

Culturing hMSCs in serum-free growth medium (MSCGM-CD or PowerStem) or serum-containing medium (MSCGM) revealed a homogenous population of fibroblast-like cells, although cell shape and size differed with culture medium. Subconfluent cells in P0 cultured in PowerStem were elongated and grew separately rather than tightly together (Figure 1). MSCGM-cultured cells appeared bigger, more spread, and with more space between individual cells in P0. In contrast, cells grown in MSCGM-CD were small and demonstrated tight growth behavior establishing denser cell fields with empty areas in between. Colony growth could also be distinguished. Although differences were not significant between groups, cells cultured in MSCGM-CD developed larger colonies and in general higher colony numbers (Figure 2) than cells grown in MSCGM or PowerStem. The average time of hMSC colonies to reach confluency in P0 was 11.5 d ± 0.96 d SEM for MSCGM, 11.3 d ± 1.97 d SEM for MSCGM-CD, and 11.8 d ± 2.23 d SEM for PowerStem (all *n* = 6). The doubling time of the cells after P1 was 2.60 d ± 0.19 d SEM for MSCGM, 2.39 d ± 0.19 d SEM for MSCGM-CD, and 4.00 d ± 2.43 d SEM for PowerStem (all *n* = 4). Since cells cultured in MSCGM-CD, and MSCGM provided highest colony numbers and most reliable growth, cells grown in these media were further analysed for surface marker expression and differentiation potential.

### 3.2. Human MSCs Cultured in Serum-Containing Medium Express Significantly More CD105 Compared to Serum-Free Cultured Cells

**Figure 3:**
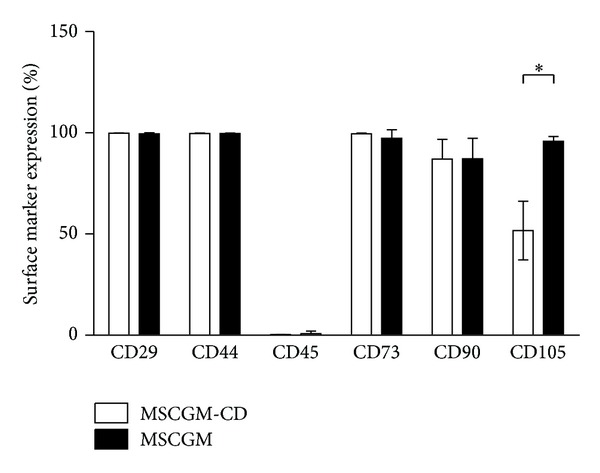
Immunophenotyping of hMSCs. MSCGM (black) as well as MSCGM-CD (white) treated cells were negative for CD45, but positive for CD29, CD90, CD44, CD73, and CD105. CD105 expression in MSCGM is significantly higher than in MSCGM-CD cultivated cells. Data presented as mean ± SEM (for CD29 expression by hMSCs grown in MSCGM *n* = 3, all other experiments *n* = 5). Unpaired two-tailed *t*-test was performed for statistical analysis. *P ≤ 0.05.

For MSCGM-CD and MSCGM growth media and each donor, the mean viability of hMSCs was always higher than 90.1% ± 5.8% SEM. Expression of the cell surface marker proteins CD29, CD44, CD45, CD73, and CD90 was similar between MSCGM and MSCGM-CD cells (Figure 3). Nearly all analyzed cells were positive for CD29, CD44, and CD73, indicating that almost all cells express the respective cell surface marker proteins (Table 2 and Figure 3). Moreover, the stem cell marker CD90 was less abundant (87.04% ± 4.39% SEM, *n* = 5 for MSCGM-CD and 87.14% ± 4.55% SEM, *n* = 5 for MSCGM), while CD45 was totally absent (Table 2 and Figure 3). Interestingly, endoglin (CD105) is present in only half of serum-free treated hMSCs compared to MSCGM-CD-cultured cells (51.70% ± 14.53% SEM, *n* = 5 in MSCGM-CD and 95.83% ± 2.35% SEM in MSCGM-cultured cells, *n* = 5, *P* ≤ 0.05). 

Growth conditions of hMSCs regarding initial colony formation, doubling time, confluency, and passage were comparable between MSCGM and MSCGM-CD. In addition, each patient's bone marrow was tested in these media in parallel. Patient cohorts were homogenous in terms of age, BMI, and cardiac condition. Likewise, none abused alcohol or tobacco. However, we cannot exclude influence on findings by former tobacco consumption (Supplementary data Figure S2). Nevertheless, Figure 2 shows that colony growth is largely dependent on the different media, whereas colony growth in each medium was highly consistent between the patients. 

### 3.3. Human MSC Cultured in Both Media Differentiated into All Three Mesenchymal Lineages

Literature data demonstrated that *ex vivo* cultivated hMSCs are able to differentiate in three different mesenchymal lineages (adipogenic, osteogenic, and chondrogenic) [9]. To check whether this is affected by serum-free conditions, hMSCs were cultured in differentiation growth medium and stimulated to commit into these lineages.

**Figure 4:**
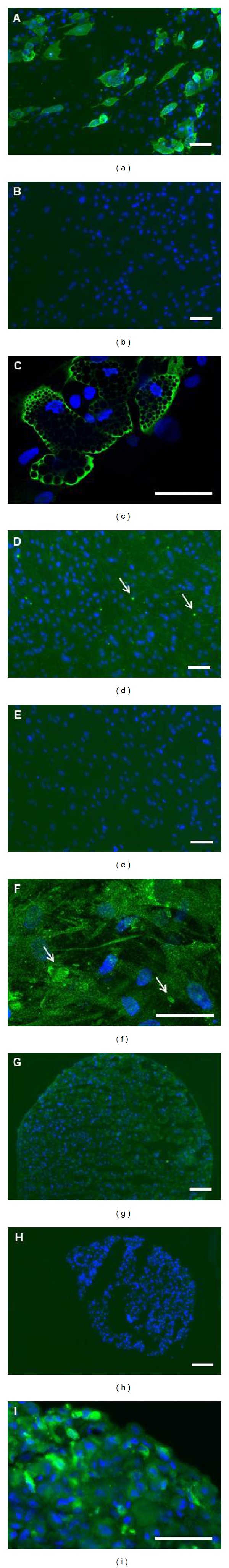
Microscopic characterization of differentiated hMSCs. Phenotypes of hMSCs after 21 days in adipogenic (a)–(c), osteogenic (d)–(f), and chondrogenic (g)–(i) differentiation culture. Images of unstained cells (b), (e), and (h) and images of immunocytochemically stained differentiated cells (a), (c), (d), (f), (g), and (i). Green color displays lineage specific proteins. Adipogenic commitment is shown by staining against fatty acid binding protein-4 (a) and (c), osteogenic commitment by antiosteocalcin staining (d) and (f), and chondrogenic differentiation by staining against aggrecan (g) and (i). Nuclei were counterstained with DAPI (blue). Pictures (b), (e), and (h) show negative controls (secondary antibody only). Arrows (d), (f) indicate accumulations of osteocalcin. These representative pictures have been taken at 100x ((a), (b), (d), (e), (g), (h), scale bar length = 100 *μ*m) and 400x ((c), (f), (i), scale bar length = 50 *μ*m) magnification.

**Figure 5:**
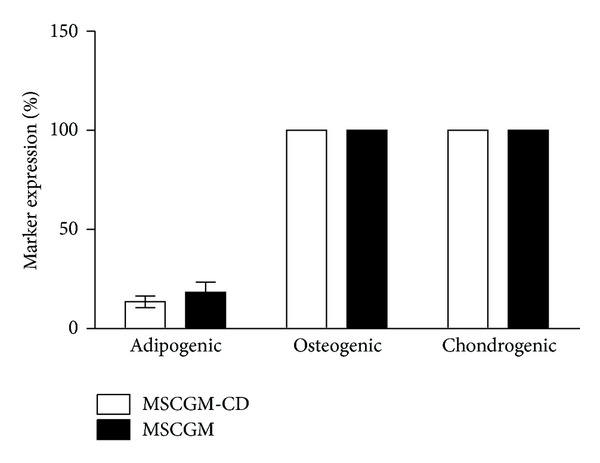
hMSC differentiation assays. MSCGM (black) as well as MSCGM-CD (white)-cultured cells revealed to be 100% positive for expression of osteogenic and chondrogenic lineage markers. Commitment along adipogenic lineage occurred in 13.51% of MSCGM-CD and in 18.37% of MSCGM propagated cells. Cells were counted in 10 high power fields that contained at least 100 cells, and the amount of nuclei (representing total amount of cells) was compared with the amount of cells positive for the differentiation marker. Data presented as mean ± SEM (*n* = 5). Unpaired two-tailed *t*-test demonstrated no significant differences between both groups.

Human MSCs differentiated in an adipocyte lineage regardless of the presence of serum within the growth medium (13.51% ± 2.89% SEM, *n* = 5 for MSCGM-CD cells compared to 18.37% ± 5.10 SEM, *n* = 5 for MSCGM; Figures 4(a), 4(c), and 5). 

Investigating hMSC differentiation into the osteogenic linage suggested an overall expression of osteocalcin (Figures 4(d) and 4(f)). If single cells could be distinguished, the antiosteocalcin green fluorescence seemed to cover whole cell bodies. This indicates that osteocalcin is at least partly accumulated in the cytoplasm or covers plasma membrane. Furthermore, several spots of brighter fluorescence representing accumulations of osteocalcin were detected (Figures 4(d) and 4(f) arrow). Thus, no alteration in differentiation between MSCGM and MSCGM-CD-cultured cells was found, as in both groups all cells correspond to the osteogenic phenotype (100% ± 0% SEM, *n* = 5; Figure 5).

To check for chondrogenic differentiation, hMSCs were analyzed for aggrecan expression. All cells were positive for aggrecan staining (Figures 4(g) and 4(i)). Due to this staining, differentiation induced hMSCs were all determined as chondrogenic cells (100% ± 0% SEM, *n* = 5 for MSCGM and MSCGM-CD cultivated cells; Figure 5). These results may be of importance concerning the clinical use of hMSCs expanded in serum-free medium.

To verify cultured bone marrow cells as hMSCs, the expression of specific cell surface marker proteins was investigated. Irrespective of the culturing medium, cells were as expected positive for CD29, CD44, CD73, and mostly CD90. The cells did not express the hematopoietic marker CD45 (Figure 3). Only for the CD105 surface marker, significant differences between both groups were present. Under MSCGM-CD culturing conditions, approximately half of the cells (51.70%) were CD105 positive, while almost all MSCGM (95.82%) treated cells were positive. This difference in CD105 expression could be caused by the composition of the growth medium. It might contain factors that can induce CD105 expression. TGF-*β* has been shown to upregulate CD105 transcription under hypoxic conditions [21]. However, since the knowledge about precise medium composition is manufactures property, this influence is to be hypothesized and needs further investigations. Previous data indicate that CD105 plays an important role in angiogenesis [12]. Our own analysis demonstrated that CD105 expression of hMSCs has a significant impact on regenerative potential in a murine model of myocardial infarction [22]. We hypothesize that this improvement might be caused by induction of proliferation or cytoprotection under hypoxic conditions of CD105^+^-cells [22]. Additionally, a murine model of cardiac ischemia revealed that transplantation of hMSCs selected for CD105 enhanced heart performance six weeks after treatment compared to unpurified hMSCs [22]. Taken together, this indicates that serum-free cultured hMSCs might lead to an impaired therapeutic outcome. *In vivo* studies, comparing hMSCs expanded in serum-free and serum-containing medium will have to be conducted to show whether the cells are equally suitable for cardiac regeneration.

In order to examine whether hMSCs maintain their plasticity during serum-free culture, they were stimulated to differentiate along adipogenic, osteogenic, and chondrogenic lineages, respectively. Adipogenic differentiation generally occurred less frequent (approximately 15% cells showed adipogenic differentiation), while markers of osteogenic and chondrogenic differentiation appeared in all cells. This could be due to the fact that already committed mesenchymal progenitor cells were present in the bone marrow cultures [23]. As Muraglia and coworkers already demonstrated, about one-third of hMSCs possess the potential to differentiate along the adipogenic lineage, whereas the majority of cells exhibit osteochondrogenic potential [23]. This corresponds to our findings (Figure 5). 

The rate of adipogenic differentiation was less than 30%, which might depend on the bone marrow aspirate itself. In our study, bone marrow donors were older than in Muraglia's study (average age in our study: 73.6 years compared to an age range of Muraglia's donors between 5 month and 30 years) [23]. Therefore, bone marrow cells might have undergone beginning senescence in our study, resulting in lower plasticity. Additionally, Muraglia used bone marrow from the iliac crest while we used sternum-derived bone marrow, and it is unknown whether this has an additional influence on differentiation potential [23]. In addition, Levi et al. demonstrated that adipose tissue-derived hMSCs which were enriched for expression of CD105^high^ have a reduced osteogenic potential as compared to CD105^low^ [24]. This may be characteristic of adipose-derived hMSCs, as evident from our results for bone marrow-derived hMSCs: in the latter, we did not observe a correlation between CD105 expression and osteogenic potential. Additionally, Levi et al. used hMSCs in P1 and induced osteogenesis for 7 days, whereas we used hMSCs in P3 and induced for 21 days. Therefore, it might be a time-dependent effect. Previous studies that used protocols similar to ours did show that the majority of adipose-tissue, bone marrow, and cord blood-derived hMSCs do share common characteristics [25] and are able to differentiate along the osteogenic lineage [26]. 

The media used for adipogenic and osteogenic differentiation contained 10% FBS. At the time this work was conducted, serum-free differentiation medium was not commercially available. Previously, serum-free cultured cells have been therefore treated with serum to induce differentiation. However, preculture under serum-free conditions did not significantly influence the ability to differentiate along these lineages. Although not statistically significant, adipogenic differentiated cells tend to display reduced differentiation for individual donors. Thus, MSCGM-CD-cultured cells might have lost adipogenic differentiation potential to a certain extent. This aspect will be investigated in more detail in further studies. 

Osteogenic and chondrogenic markers expression was observed for all cells according to the induced lineage. Although osteocalcin and aggrecan are proteins of the extracellular matrix, they are also present intracellularly, probably due to accumulation prior to secretion. In both lineages, cells grew in more than one layer. Therefore, it is possible that not every single cell differentiated, but the presence of osteocalcin and aggrecan in the extracellular space did not allow the identification of undifferentiated cells. However, it has been reported that the majority of hMSCs have the ability to differentiate into osteogenic and chondrogenic lineage [23]. Finally, MSCGM and MSCGM-CD-cultured cells displayed comparable levels of osteogenic and chondrogenic markers expression.

## 4. Conclusions

The aim of this study was to analyze proliferation and differentiation of hMSCs cultured in chemically defined, serum-free growth medium compared to conventional, serum-containing medium under comparable conditions considering cell growth, passage, and confluency. We could show that serum-free cultured hMSCs (i) express typical hMSC surface marker proteins and (ii) maintain multilineage potential. However, serum-free expanded hMSCs (iii) display a significantly lower amount of CD105 protein compared to cells grown in presence of serum (51.70% ± 14.53% SEM. *n* = 5 in MSCGM-CD and 95.83% ± 2.35% SEM in MSCGM-cultured cells, *n* = 5, *P* ≤ 0.05).

Due to their plasticity, hMSCs provide various therapy approaches in regenerative medicine, such as treatment of genetic bone disorders or cardiac remodeling after myocardial infarction. Moreover, it is very likely that further fields of application will arise in the future, and the necessity to culture and expand hMSCs *ex vivo* will increase. Since serum-containing culture media are clinically unsafe and inconstant in their formulation, complete chemically defined media are needed. To date, there is a lack of detailed studies on serum-free hMSC culturing. Our findings demonstrate that serum-free cultured hMSCs can be classified as equivalent to cells cultured in conventional medium with minor restrictions. The results of our analysis could help to introduce serum-free hMSC culture conditions to preclinical research and future therapeutic applications.

## Figures and Tables

**Table 1:** Antibodies for analytical fluorescence-activated cell sorting.

Antibody/dye	Conjugate	Company	Volume antibody*	Isotype	Company	Volume isotype*
CD29	APC	BD Biosciences	20 *μ*L	mouse IgG1 *κ*	BD Biosciences	20 *μ*L
CD44	PerCP-Cy5.5	BD Biosciences	5 *μ*L	mouse IgG2b *κ*	BD Biosciences	20 *μ*L
CD45	V500	BD Biosciences	5 *μ*L	mouse IgG1 *κ*	BD Biosciences	5 *μ*L
CD73	PE	BD Biosciences	20 *μ*L	mouse IgG1 *κ*	BD Biosciences	20 *μ*L
CD90-biotin	Streptavidin-V450	BD Biosciences	3 *μ*L	mouse IgG1 *κ*	BD Biosciences	21 *μ*L
CD105	Alexa Fluor 488	AbD Serotec	10 *μ*L	mouse IgG1	AbD Serotec	10 *μ*L
NearIR (live/dead)	—	Invitrogen	0.5 *μ*L	—	—	—

*Volumes required for up to 500,000 cells in 100 *μ*L.

APC (Allophycocyanin), PerCP-Cy5.5 (Peridinin chlorophyll protein-cyanine 5.5 conjugate), V500 (BD Horizon V500), PE (phycoerythrin), V450 (BD Horizon V450), NearIR (LIVE/DEAD Fixable Near-IR Dead Cell Stain).

**Table 2:** Human MSC surface marker expression.

Growth medium	Result	CD29^+^	CD90^+^	CD44^+^	CD45^+^	CD73^+^	CD105^+^
MSCGM-CD	mean	0.9984*	0.8704	0.9969	0.0025	0.9960	0.5170
	SEM	0.0003	0.0439	0.0010	0.0005	0.0016	0.1453
MSCGM	mean	0.9961	0.8714	0.9968	0.0080	0.9726	0.9583
	SEM	0.0021	0.0455	0.0010	0.0058	0.0192	0.0235

*n = 3, all other experiments *n* = 5, all values in %.

SEM (standard error of the mean).
